# Comparison of Cerebral Blood Flow During General Anesthesia in Elderly Patients with and Without Dementia: A Prospective Controlled Clinical Trial

**DOI:** 10.3390/jcm14196692

**Published:** 2025-09-23

**Authors:** Yoshinari Morimoto, Megumi Hayashi, Yohei Tanaka, Hitomi Nishizaki, Masayoshi Shirakawa, Ryota Tamura, Lou Mikuzuki

**Affiliations:** Department of Geriatric Dentistry, Kanagawa Dental University, 82 Inaoka-cho, Yokosuka 238-8580, Japan

**Keywords:** cerebral blood flow, dementia, general anesthesia, near-infrared spectroscopy

## Abstract

**Background/Objectives**: The maintenance of cerebral blood flow (CBF) by managing blood pressure and brain cell activity and avoiding hypocapnia is important when administering anesthesia to patients with dementia. This study aimed to evaluate CBF during general anesthesia in elderly patients with severe dementia while maintaining their physiological parameters within an adequate range. **Methods**: The patients were anesthetized within a set range of parameters without affecting CBF (mean arterial pressure [MAP] > 50 mmHg; bispectral index [BIS] > 20; percutaneous arterial oxygen saturation [SpO_2_] > 95%; end-tidal CO_2_ [etCO_2_] 35–40 mmHg). The normalized tissue hemoglobin index (nTHI), which reflects CBF, was measured using near-infrared spectroscopy. The parameters were compared between patients with severe dementia (*n* = 13) and those without cognitive impairment (*n* = 13). **Results**: There were no differences in patient background. A similar decline in MAP and BIS values was observed in both groups, but the values remained within the set range. The nTHI decreased significantly to 0.60 in the dementia group and to 0.79 in the non-dementia group after the start of the treatment (*p* ≤ 0.049). Even when the MAP, BIS, SpO_2_, and etCO_2_ values were maintained in their adequate ranges during general anesthesia, the nTHI decreased by 40% in the dementia group. **Conclusions**: These findings indicate that CBF greatly decreases in elderly patients with severe dementia during general anesthesia.

## 1. Introduction

General anesthesia is used for behavior management during dental treatment for patients with advanced dementia when they show resistance or refuse to undergo treatment [1,2,3]. Cognitive impairments that develop after surgery are classified as postoperative delirium (POD) and postoperative cognitive dysfunction (POCD) [4,5,6].

Clinical studies have investigated the effects of anesthetics and sedatives on cognitive function. No difference in the incidence of POD or POCD has been observed with either general or local anesthesia [7], anesthesia with volatile anesthetic or with propofol [8], and the volatile anesthetics sevoflurane or desflurane [9]. Clinical studies that used a clinical database to examine the history of general anesthesia in patients who developed dementia did not find any difference in the presence or absence of exposure to general anesthesia or the number of exposures; the relationship between the onset of dementia and general anesthesia is unclear [10,11,12,13,14,15,16,17,18]. Meanwhile, retrospective clinical studies in the orthopedics field have shown that general anesthesia is associated with the progression of dementia and with the complications in the perioperative period in patients with dementia [12,19,20,21]. A study also reported that patients with dementia typically require long hospital stays [21]. However, these were retrospective studies based on database and clinical results on the use of general anesthesia in patients diagnosed with dementia, which did not prospectively assess the details of cognitive impairments or effects of anesthetics and sedatives.

In basic research using neurons, in vivo studies using mice and rats have shown that volatile anesthetics (sevoflurane, isoflurane) increase the accumulation of the amyloid β protein and the promotion of the phosphorylation of the τ protein [22,23,24,25,26,27,28,29,30]. In an in vitro study using cultured neurons, the intravenous anesthetics propofol and thiopental suppress the accumulation of these proteins [31,32]. However, regarding midazolam, the findings are contradictory, with one study reporting that the accumulation of these proteins is inhibited by midazolam [32] and another study reporting that it is enhanced by midazolam [33]. Reports from both clinical and basic research are therefore conflicting, and no definite conclusion has been reached.

Maintaining neuronal activity (brainwaves and cerebral blood flow [CBF]) while under anesthesia is important for maintaining perioperative cognitive function [4,5]. Hence, maintaining the bispectral index [BIS] value and tissue oxygen partial pressure at adequate levels and avoiding hypotension and hypocapnia are important. However, it is unclear whether CBF can be maintained in elderly patients with dementia even when these physiological factors are maintained adequately during anesthesia [4,5]. No prospective clinical studies have evaluated the effects of anesthetics on brain cell activity in elderly patients with severe dementia.

The objective of this study was to prospectively investigate and compare the effects of anesthesia on CBF between elderly individuals with severe dementia and those without cognitive impairment. Elderly patients with severe dementia who were scheduled to undergo behavior management via general anesthesia during dental treatment were prospectively enrolled. Anesthesia was performed with the physiological parameters (mean arterial pressure [MAP], BIS, percutaneous arterial oxygen saturation [SpO_2_], and end-tidal CO_2_ [etCO_2_]) maintained in an adequate range.

## 2. Materials and Methods

### 2.1. Study Design and Ethics Declarations

This prospective controlled clinical trial was conducted in accordance with the Helsinki Declaration. The study protocol was approved by the Institutional Research Board and Ethics Committee of Kanagawa Dental University (approval number 472) on 30 November 2017. The procedures, benefits, safety, and risks were thoroughly explained to each participant and/or guardian, and written informed consent was obtained. The research protocol was registered and published at the University Hospital Medical Information Network Center (UMIN Clinical Trials Registry) (ID: UMIN000037957, registered on 7 September 2019). We referred to our previous articles regarding the patient selection criteria, measurements, and statistical analyses in this study [34,35].

### 2.2. Study Participants

The participants were patients with severe dementia aged 60 years and older who visited the geriatric dental clinic at Kanagawa Dental University Hospital between September 2019 and March 2025 and were classified as physical status (PS) class 1 or 2 of the American Society of Anesthesiologists (ASA) classification except for severe dementia. Of these patients, those who were scheduled to undergo dental treatment (including dental extraction) using general anesthesia for specific reasons, including being uncooperative to undergo dental treatment due to advanced dementia, dental phobia, and a severe gagging reflex without cognitive impairment, were selected as potential candidates.

Once the schedule of general anesthesia was decided, a Mini Mental State Examination (MMSE) was performed. Participants with an MMSE score ≥ 24 were considered as “not having a cognitive impairment,” and they were included in the “dementia-negative group” [35]. Conversely, those with an MMSE score ≤ 23 or those who could not undergo the MMSE due to cognitive decline were considered as having dementia [36]. Of the elderly patients with dementia, those who met the following criteria were considered to have “severe dementia” and were included in the “dementia-positive group”: patients with Alzheimer-type dementia with Functional Assessment Staging of Alzheimer’s disease (FAST) stage 6 or 7; patients classified by the Clinical Dementia Rating (CDR) as having “severe” dementia. The MMSE, FAST, and CDR were assessed by medical specialists other than the attending anesthesiologists. These parameters were also evaluated a day after the general anesthesia. The following patients were excluded: those without severe dementia (MMSE ≤ 23 but with FAST ≤ 5 and/or CDR ≤ 2); those with a history of cerebrovascular disease (including cerebrovascular dementia); those with an ASA PS classification level 3 or higher. In Japan, anti-dementia agents are often not prescribed to patients with severe dementia because they have little effect on them, and pose a risk of side effects (dizziness, unsteadiness, etc.). No patients with severe dementia thus received any anti-dementia agents.

**Figure 1:**
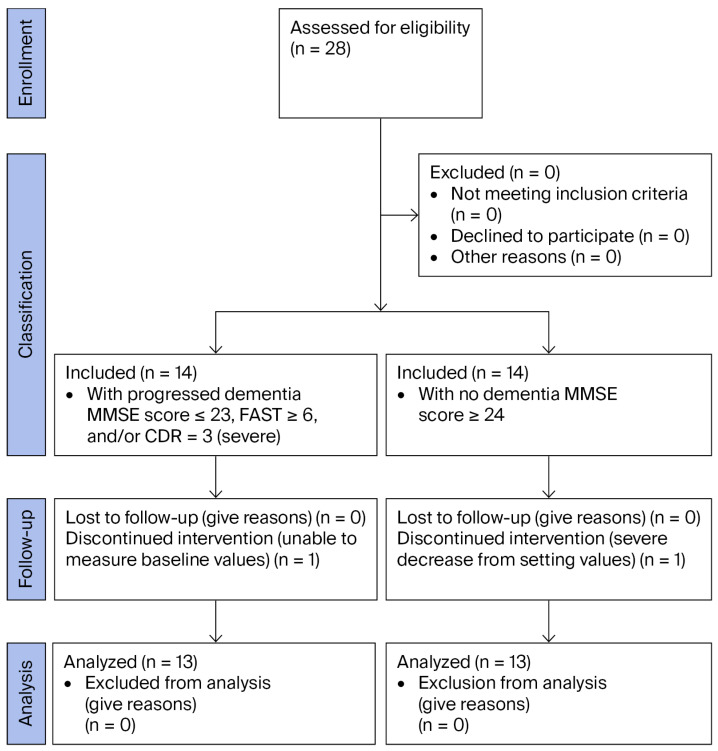
Flowchart of the patient selection process.

The study procedures were explained to all patients who met the above-mentioned inclusion criteria and/or their guardians during the study period. A total of 28 patients (14 in each group) who provided consent were included. However, one patient in the dementia-positive group was excluded because the patient was hyperactive, and thus, the baseline values could not be measured. One patient in the dementia-negative group was excluded because their MAP values fell below the lowest values set during anesthesia induction. Finally, 26 patients were enrolled, with 13 in the dementia-positive group and 13 in the dementia-negative group (Figure 1).

### 2.3. Measurements

#### 2.3.1. Pretreatment Fasting

Based on the “Practice Guidelines for Preoperative Fasting” published by the Japanese Society of Anesthesiologists, the participants were asked to refrain from eating and drinking water 8 and 2 h, respectively, before the start of anesthesia [37].

#### 2.3.2. Monitoring and Anesthesia Method

No preanesthetic medications were used. The participants entered the treatment room at 9:30 am. After entering the treatment room, the patient was placed in a horizontal position on the treatment table. A non-invasive blood pressure monitor, electrocardiograph, and an SpO_2_ monitor were attached, and a BIS sensor and a near-infrared spectroscopy (NIRS) (NIRO^®^-200NX; Hamamatsu Photonics Co., Hamamatsu, Japan) probe were placed on the forehead [3].

A peripheral intravenous line was secured using a 22-gauge intravenous cannula at the dorsum of the right or left hand. Ringer’s acetate solution (6 mL/kg/h 1% glucose and Ringer’s acetate solution; Fisio 140^®^; Otsuka Co., Tokyo, Japan) was delivered.

General anesthesia was induced slowly with a gradual increase of sevoflurane concentration from 2% to the adequate level (up to 5%) every few minutes in 6 L/min oxygen. Fentanyl 25 µg was administered several times (at least 2 min between doses), or the continuous administration of remifentanil 0.05–0.15 µg/kg/min was started. Mask ventilation with 6 L/min oxygen and 2–5% sevoflurane was provided for 3 min or more, followed by 0.6 mg/kg of rocuronium bromide, and tracheal intubation was performed after confirming that the BIS value was ≤60. After intubation, anesthesia was maintained with oxygen 1 L/min, air 5 L/min, 0.5–1.5% sevoflurane and fentanyl (25 μg, each administration), and/or remifentanil (0.05–0.15 μg/kg/min), as needed. During anesthesia induction and maintenance, the sevoflurane, fentanyl, and remifentanil doses were adjusted to maintain MAP ≥ 50 mmHg and BIS ≥ 20 (lowest values set). The patients whose measurement values fell below the lowest value set were excluded from the study procedure. Ventilator settings were adjusted to achieve a tidal volume of 6–9 mL/kg per breath and 10–14 respirations per minute. Respiration was controlled to achieve etCO_2_ concentration of 35–40 mmHg. A rectal temperature of 36–37 °C was maintained throughout the treatment.

All participants were transnasally intubated. After anesthesia was induced, left and right nostril patency was determined using a cotton swab during mask ventilation. Approximately 1.0 mL of 4% lidocaine hydrochloride spray was sprayed into the more patent nostril to achieve topical anesthesia. An Ivory PVC, Nasal, Soft Seal^®^ Cuff Tracheal Tube (Smith Medical Japan Ltd., Tokyo, Japan) (inner diameter: men, 6.5 mm; women, 6.0 mm) was inserted into the selected nostril. Only anesthesiologists with 5 years or more of experience were involved in the study.

#### 2.3.3. Measurement Items and Timing

Age, sex, height, weight, and body mass index (BMI) were extracted from medical records as patient background data. Regarding general anesthesia, the treatment time, anesthesia time, sevoflurane concentration, and the amount of fentanyl and remifentanil during both induction and maintenance were noted. The parameters measured under anesthesia were the MAP, SpO_2_, etCO_2_, and BIS values, and NIRO^®^ measurement values (right and left normalized tissue hemoglobin index [nTHI] and tissue oxygenation index [TOI]) [38].

As for the measurement times during anesthesia, MAP, SpO_2_, BIS, nTHI, and TOI were assessed at four measurement points: 5 min after the start of measurement, immediately before anesthesia induction (period of stabilizing the measurements) (MAP 1, SpO_2_ 1, BIS 1, nTHI 1, and TOI 1); period from anesthesia induction to immediately before tracheal intubation (MAP 2, SpO_2_ 2, BIS 2, nTHI 2, and TOI 2); period from tracheal intubation to immediately before treatment initiation (MAP 3, SpO_2_ 3, BIS 3, nTHI 3, and TOI 3); during the 20 min period after starting the treatment (MAP 4, SpO_2_ 4, BIS 4, nTHI 4, and TOI 4). The etCO_2_ value was measured immediately after tracheal intubation (etCO_2_ 1) and during the 20 min period after the start of treatment (etCO_2_ 2), and the highest and lowest values of each parameter were extracted. The treatment contents for the participants were intraoral dental X-ray measurement and scaling and tooth cleaning for the first 20 min or more, and since the treatment invasion is considered constant, the parameters for this period were extracted as data after the start of treatment. The treatment was started 10–20 min after tracheal intubation.

When measuring with a NIRO^®^-200NX, care was taken to ensure that none of the participants had anemia with a hemoglobin level < 10 g/dL, that areas with cutaneous veins are avoided when placing the probes on the forehead, and that measurements were obtained after the participants rested for 5 min in an horizontal position [34,35,38].

### 2.4. Statistical Analyses

Statistical analyses were performed using SPSS version 16.0 (SPSS Japan, Tokyo, Japan). Data are presented as median (interquartile range) values. The chi-squared test was used to assess sex differences, and the Mann–Whitney U test was used to conduct comparisons of other parameters between the dementia-positive and dementia-negative groups. For the intragroup comparisons, the Friedman test was used for the MAP, SpO_2_, BIS, nTHI, and TOI values, and the Wilcoxon signed rank test with Bonferroni’s correction was used for post hoc analysis. The Wilcoxon signed rank test was used for the etCO_2_ values, as they were before and after comparisons. Significance was set at *p* < 0.05 (*p* < 0.0083 after Bonferroni’s correction).

The primary end points were nTHI and TOI. The required number of cases was determined based on our previous reports [34,35,39]. Based on the sample size calculation, the final required number of patients was 14 in each group (28 in total) (Figure 1).

Prior to this study, a preliminary study was conducted on 4 patients with dementia and 4 patients without dementia. At that time, when anesthesia was rapidly induced with propofol 1–1.5 mg/kg and sevoflurane 5%, the BIS value decreased to 20 or less in four patients with or without dementia, and one patient with dementia had a MAP < 50 mmHg. Anesthesia was therefore induced slowly, and the amount of anesthetic was adjusted to maintain a BIS value ≥ 20 and MAP ≥ 50 mmHg.

## 3. Results

### 3.1. Patients’ Background Characteristics

A total of 26 elderly patients (16 men and 10 women) with a median age of 70 (quartile, 62.8–77.8) years, median height of 158.4 (153.3–165.8) cm, median weight of 50.4 (45.1–56.3) kg, and median BMI of 20.4 (19.3–22.6) kg/m^2^ participated in the study. The dementia-positive group consisted of 11 patients with Alzheimer-type dementia and 2 patients with Lewy body dementia. The FAST stages of the 11 patients with Alzheimer-type dementia were as follows: stage 6e, 1 patient; stage 7a, 2 patients; stage 7b, 7 patients; stage 7c, 1 patient. The CDR for all these patients was “severe.” The dementia-negative group consisted of 11 patients with a dental phobia and 2 patients with a severe gagging reflex. None of the dementia-positive patients were taking anti-dementia drugs. The median treatment time was 167 (146.3–190.5) min, and the median anesthesia time was 225 (194.8–243.3) min.

### 3.2. Comparison of Patients’ Characteristics and Vital Signs

No differences in age, sex, height, weight, or BMI values were observed between the dementia-positive and dementia-negative groups. The treatment time (*p* = 0.029) and anesthesia time (*p* = 0.016) were significantly longer in the dementia-positive group, but this was not considered to affect the results of the present study (Table 1). No differences were observed in the sevoflurane concentrations and fentanyl or remifentanil dose between the two groups (Table 1).

**Figure 2:**
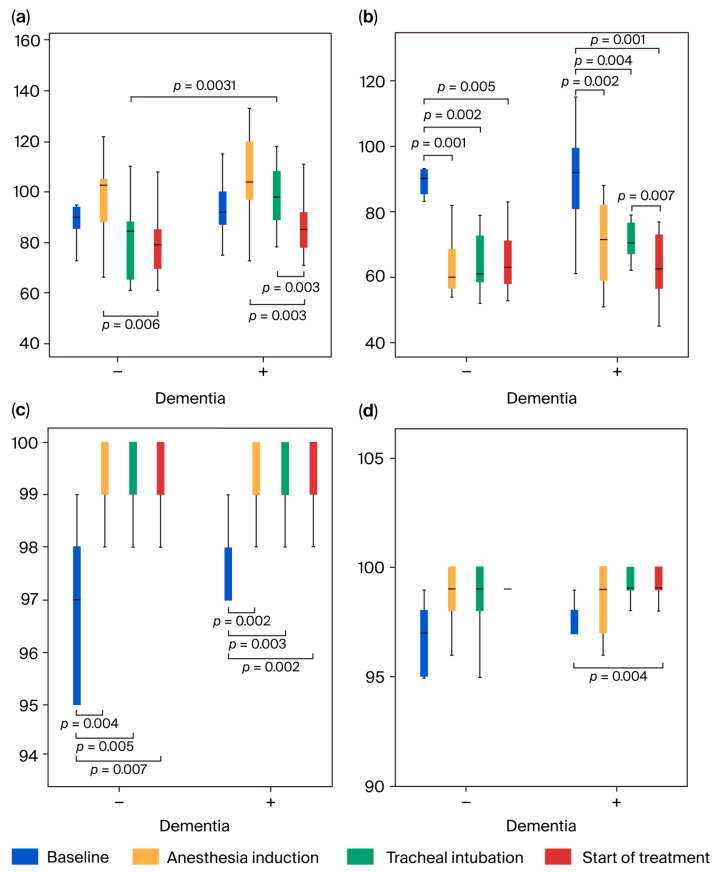
Changes in the mean arterial pressure (MAP; (**a**,**b**)) and percutaneous arterial oxygen saturation (SpO_2_; (**c**,**d**)). Measurement timings: 5 min after the start of measurement (immediately before anesthesia induction; baseline); from anesthesia induction to immediately before tracheal intubation; from tracheal intubation to immediately before treatment initiation; during 20 min after start of treatment. (**a**) Maximum MAP values and (**b**) minimum MAP values: the maximum MAP values indicate significant difference among the four measurement points in both groups (*p* = 0.003), and post hoc analysis was performed (indicated in the figure). The minimum MAP values indicate a significant difference among the four measurement points in both groups (*p* = 0.001), and post hoc analysis was performed (indicated in the figure). (**c**) Maximum SpO_2_ values and (**d**) minimum SpO_2_ values: the maximum and minimum SpO_2_ values indicate a significant difference among the four measurement points in both groups (*p* ≤ 0.002), and post hoc analysis was performed (indicated in the figure). The data in Figure 2, Figure 3, Figure 4 and Figure 5. are suggested in Tables S1 and S2.

**Figure 3:**
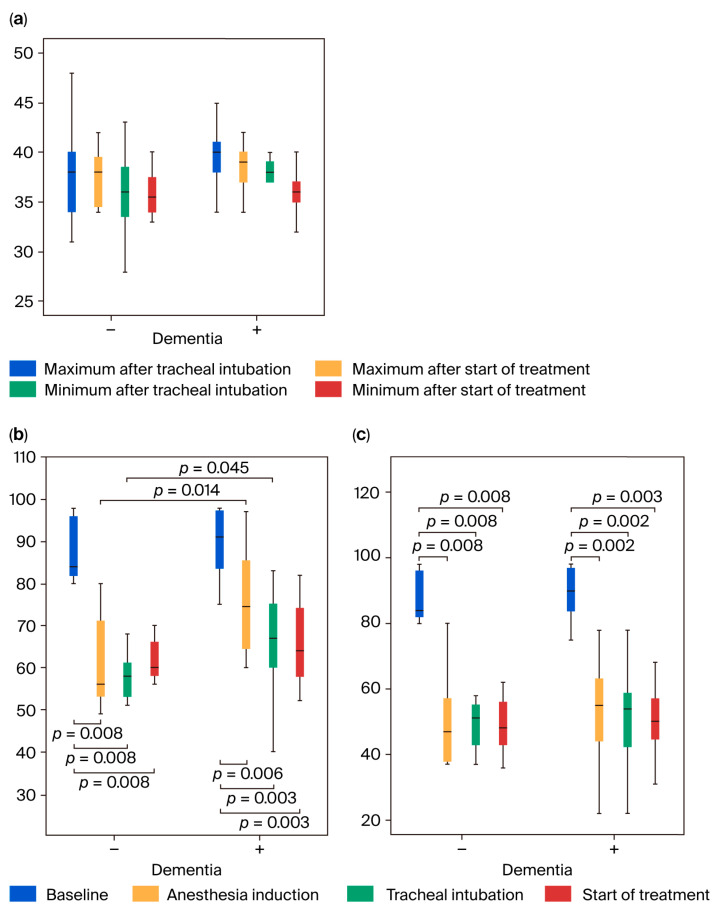
Changes in end-tidal CO_2_ (etCO_2_; (**a**)) and bispectral index (BIS) values (**b**,**c**). (**a**) Measurement timings: from tracheal intubation to immediately before treatment initiation; during 20 min after start of treatment. The left 2 bars indicate maximum data and the right 2 bars indicate minimum data in both groups, respectively. The measurement timings for (**b**,**c**) are the same as in Figure 2. (**b**) Maximum BIS values and (**c**) minimum BIS values: the maximum and minimum BIS values indicate a significant difference among the four measurement points in both groups (*p* ≤ 0.001), and post hoc analysis was performed (indicated in the figure).

The highest MAP 3 value was significantly higher in the dementia-positive group than in the dementia-negative group (*p* = 0.0031; Figure 2a,b). No differences in SpO_2_ or etCO_2_ were observed between the two groups at all measurement points (Figure 2c,d and Figure 3a). The highest BIS values of BIS 2 and 3 were significantly higher in the dementia-positive group than in the dementia-negative group (*p* ≤ 0.045), but no difference was observed in the lowest BIS values between both groups (Figure 3b,c).

Regarding intragroup comparison, the highest values of MAP 4 significantly decreased compared with MAP 2 in the dementia-negative group (*p* = 0.006), and the highest values of MAP 3 and MAP 4 significantly decreased compared with MAP 2 in the dementia-positive group (*p* = 0.003). The lowest values of MAP 2, 3, and 4 significantly decreased compared with MAP 1 in both groups (*p* ≤ 0.005). The lowest value was 60 mmHg (median) or higher (Figure 2a,b). The highest value of SpO_2_ was significantly increased at SpO_2_ 2, 3, and 4 compared with SpO_2_ 1 in both groups (*p* ≤ 0.007), and the lowest value was significantly increased at SpO_2_ 4 compared with SpO_2_ 1 in the dementia-positive group (*p* = 0.004). The increase in SpO_2_ from 97% (before induction) to 99–100% (after induction) was due to oxygen administration (Figure 2c,d). There was no significant difference in the highest and lowest values of etCO_2_ between the two measurement points in both groups, and the median values were both within the range of 37–39 mmHg, proving that adequate ventilation was maintained (Figure 3a). In both groups, the highest and lowest BIS values of BIS 2, 3, and 4 decreased significantly compared with BIS 1 (*p* ≤ 0.008), and the lowest values were 44.5 (median) or higher (Figure 3b,c).

### 3.3. NIRS Measurement Parameters

#### 3.3.1. Change in nTHI

**Figure 4:**
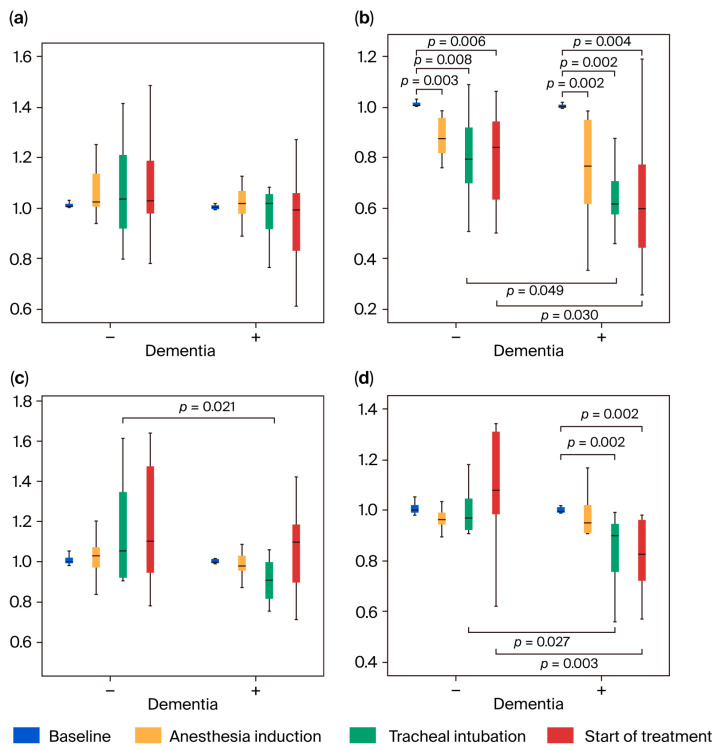
Changes in the normalized tissue hemoglobin index (nTHI). The measurement timings are the same as in Figure 2. (**a**) Maximum right nTHI values, (**b**) minimum right nTHI values, (**c**) maximum left nTHI values, and (**d**) minimum left nTHI values: the minimum values of the right nTHI indicate a significant difference among the four measurement points in the dementia-positive group (*p* = 0.001), and post hoc analysis was performed (indicated in the figure). The minimum values of the left nTHI indicate significant difference among the four measurement points in the dementia-positive group (*p* < 0.001), and post hoc analysis was performed (indicated in the figure). The minimum values of the right nTHI 3 and 4 and left nTHI 3 and 4 were significantly lower in the dementia-positive group than those in the dementia-negative group, respectively (*p* ≤ 0.049).

The lowest values of right nTHI (rnTHI) 3 and 4 and left nTHI (lnTHI) 3 and 4 were significantly lower in the dementia-positive group than those in the dementia-negative group (*p* ≤ 0.049; Figure 4a,b). The highest values of lnTHI 3 were significantly lower in the dementia-positive group than those in the dementia-negative group (*p* = 0.021; Figure 4c,d).

Regarding intragroup comparison, the measurement of the nTHI at the reference point (nTHI 1 on the right [rnTHI 1] and nTHI 1 on the left [lnTHI 1]) showed the baseline value (1) in both groups. However, at the highest nTHI level in the dementia-positive group, there was no significant change in either group. In the lowest nTHI value, rnTHI 2, 3, and 4 were 0.77, 0.61, and 0.60, respectively, and rnTHI 2, 3, and 4 were significantly lower than rnTHI 1 (*p* ≤ 0.004). The lowest values of lnTHI 2, 3, and 4 were 0.95, 0.90, and 0.85, respectively, and lnTHI 3 and 4 were significantly lower than lnTHI 1 in the dementia-positive group (*p* = 0.002) (Figure 4a–d). Meanwhile, at the lowest nTHI value in the dementia-negative group, rnTHI 2, 3, and 4 were 0.87, 0.79, and 0.84, respectively, and rnTHI 2, 3, and 4 were significantly lower than rnTHI 1 (*p* ≤ 0.008). No difference was observed in the lowest values of lnTHI 2, 3, and 4. The decline in nTHI was at most −40% in the dementia-positive group, whereas it remained at −20% in the dementia-negative group (Figure 4a–d).

#### 3.3.2. Changes in TOI

**Figure 5:**
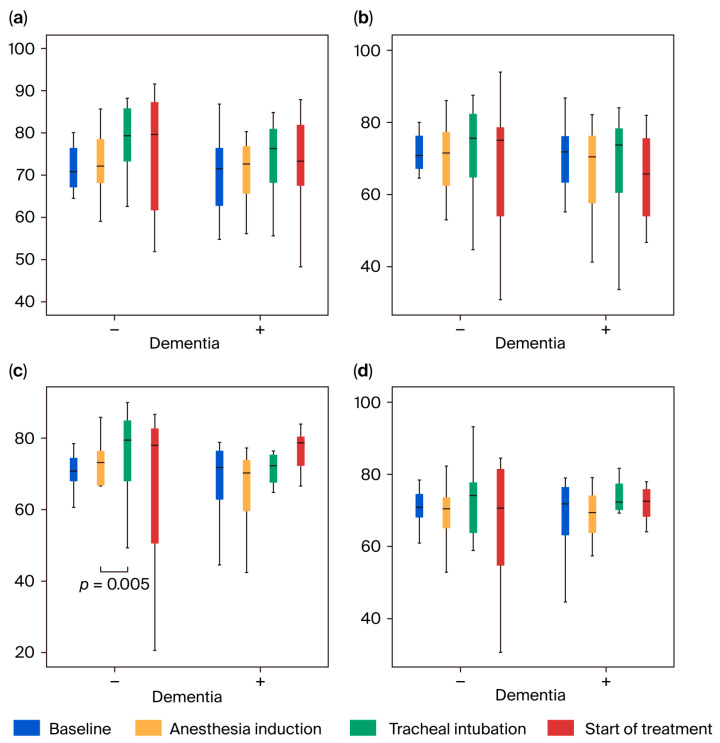
Changes in the tissue oxygenation index (TOI). The measurement timings are the same as in Figure 2. (**a**) Maximum right TOI values, (**b**) minimum right TOI values, (**c**) maximum left TOI values, and (**d**) minimum left TOI values; the results suggest a significant difference between the maximum left TOI 2 and left TOI 3 values in the dementia-negative group (*p* = 0.005).

There was no difference in TOI changes between the dementia-positive and dementia-negative groups at each measurement point. In the comparison within each group, there was a significant difference in left TOI (lTOI) 2 and lTOI 3 in the dementia-negative group (*p* = 0.005), but no differences were observed within the two groups at any other measurement point (Figure 5a–d).

### 3.4. Changes in Cognitive Function

The cognitive function of the patients in the dementia-positive group was evaluated using the FAST and CDR before and the day after general anesthesia, and none of the patients showed cognitive decline.

## 4. Discussion

This study demonstrated that when the anesthesia was induced slowly and maintained without an excessive decrease in MAP, SpO_2_, and BIS values and with an adequate etCO_2_ adjustment, the nTHI (which reflects the CBF) decreased by 40% in elderly patients with severe dementia after the start of treatment, indicating significantly lower nTHI values than in elderly patients with no cognitive decline (−20%). No change was observed in the TOI in either group.

CBF is physiologically maintained in the constant range of MAP 60–150 mmHg under consciousness (the autoregulation of CBF) [40]. CBF increases by 1 to 2 mL/100 g/min for each 1 mmHg increase in PaCO_2_ around normal PaCO_2_ values (PaCO_2_ range, 25–70 mmHg) [40]. Furthermore, CBF increases rapidly when PaO_2_ < 60 mmHg. Meanwhile, 60% of the brain’s energy expenditure contributes to electrophysiological functions, and the cerebral metabolic rate (CMR) is related to CBF [41]. MAP and CBF (middle cerebral artery flow velocity) have been reported to be correlated during anesthesia in a study of patients with an average age of 51 years [42], and that in the anesthesia using propofol (1–2 mg/kg), remifentanil (anesthesia induction with 1 μg/kg/min and maintenance using 0.1 μg/kg/min), and sevoflurane (<1 minimum alveolar concentration [MAC]), the MAP and degree of expansion of the internal carotid and middle cerebral arteries are correlated [43]. These reports suggest that MAP and CBF are correlated even under anesthesia. In this study, the set of vital signs with the lowest values during anesthesia (avoiding MAP < 50 mmHg and BIS < 20) were determined as a range that does not affect CBF based on these physiological data. In the present study, the lowest MAP value was 60 mmHg (median value), which is considered to be within the category of the autoregulation of CBF. The PaCO_2_ of 37–39 mmHg (median value) and SpO_2_ of 97–100% (equivalent to PaO_2_ > 80 mmHg; median value) are considered to have small physiological effect on CBF. The lowest value of BIS is 44.5 (median value), which is appropriate for general anesthesia without suppressing electrophysiological functions excessively.

When a volatile anesthetic is administered, CBF is determined by the balance between CBF reduction due to CMR suppression and CBF increase due to direct cerebral vasodilation. When the volatile anesthetic is 0.5 MAC, CBF decreases because CMR suppression becomes dominant. However, at 1 MAC, both are antagonistic and CBF does not change, but when it exceeds 1 MAC, vasodilation becomes dominant and CBF increases significantly [41]. In this study, both groups had >1 MAC in sevoflurane concentration (5.0%, median value), and MAP > 60 mmHg was maintained during anesthesia induction. Although CBF should be physiologically in a state of increase, both groups showed a decrease in CBF. Furthermore, during anesthesia maintenance, the sevoflurane concentration (median value) in both groups was 0.5–1.5%, <1 MAC, and MAP > 60 mmHg; physiologically, the CBF should thus remain unchanged. However, again, both groups showed a decrease in CBF.

Regarding intravenous anesthetics and narcotics, during anesthesia induction, fentanyl 75 μg (median) and remifentanil 0.2 μg/kg/min (median) were used in both groups. During the maintenance of anesthesia, the dementia-positive group received 25 μg of fentanyl and 0.06–0.1 μg/kg/min of remifentanil, and the dementia-negative group received 50 μg of fentanyl and 0.1–0.15 μg/kg/min of remifentanil, with no significant difference. Propofol, which was used in the pilot study, decreased CBF by 53–79%, potentially affecting the results, and it was therefore not used in the present study [41,44]. Some studies reported no change in CBF and the cerebral metabolic rate of oxygen (CMRO_2_) when fentanyl was used at an average dose of 16 μg/kg, and other studies reported that CBF increased in the frontal, temporal, and cerebellum areas when used at 1.5 μg/kg, but decreased in other areas. Meanwhile, remifentanil at 0.05–0.15 μg/kg/min causes a mild increase in CBF in the frontal, inferior parietal lobule and motor cortex region, and a decrease in the cerebellum, superior temporal gyrus, and periaqueductal gray matter [41,45]. As the CBF was measured in this study using a probe on the forehead, both fentanyl and remifentanil at the doses used in this study should physiologically indicate a slight increase in CBF on the forehead. However, both groups showed a decrease in CBF.

In a report on anesthesia and CBF in patients without dementia, Soejima et al. reported that with anesthesia using remifentanil (0.1–0.3 μg/kg/min) and sevoflurane (1–2%), MAP decreased to approximately 60 mmHg (−25%) and CBF was −20% from the baseline, consistent with the present study [46]. The following factors concerning CBF reduction are considered: MAP has decreased to the lower limit of CBF autoregulation; CBF may have decreased due to propofol [46]. In other studies in patients with an average age of 47 years, anesthesia using propofol and remifentanil decreased both MAP (−17% to 34%) and CBF (blood velocity of the middle cerebral artery: Vm, −28% to 39%) [47], and another study showed that CBF decreased to 81% at 0.75 MAC of sevoflurane, 74% at 1 MAC, and 79% at 1.25 MAC compared with the baseline (before sevoflurane administration) [48]. Based on these results, CBF decreases by approximately 20–25% due to general anesthesia. Even in elderly people older than 65 years, the upper and lower limits of MAP at which CBF becomes constant do not change at a sevoflurane concentration of 1 MAC [49], and in another study of patients with an average age of 37 years, CBF autoregulation and CO_2_ reactivity are maintained at a sevoflurane concentration of 1 MAC [50].

Even in the patients without cognitive decline in this study, CBF decreased by −20% due to anesthesia, which is similar to a previous report [46]. In this study, there was less significant difference in the dose of anesthetics or opioids between the two groups. CBF should physiologically increase with sevoflurane of 1 MAC or more at the time of anesthesia induction, or CBF should stay constant during anesthesia maintenance with 0.5 MAC of sevoflurane, fentanyl, and remifentanil. However, it is unlikely that CBF decreases. Furthermore, because the type and amount of anesthetics used were adjusted in this study, both elderly patients with dementia and those without cognitive decline showed similar trends in the MAP, BIS, SpO_2_, and etCO_2_ values. When MAP decreased to the lower limit of CBF autoregulation in the patients aged approximately 70 years, CBF could therefore decrease in both groups. However, the degree of decline is greater in patients with severe dementia; elderly patients with severe dementia might have had a weakened CBF autoregulation mechanism or an already-decreased brain tissue metabolism.

Compared with these traditional CBF biomarkers, the multidomain features of a pulse wave can provide more comprehensive information about cerebral conditions. The photoplethysmography (PPG) waveform features reflect the volumetric changes in the distal circulation and detect different aspects of balanced general anesthesia [51]. Zhang et al. and Chen et al. stated that the PPG-derived parameters are related to remifentanil concentration and more suitable in monitoring the nociceptive component in the balanced general anesthesia, while the cerebral state index (CSI)—derived from four sub-parameters of the electroencephalogram—performs well in detecting the sedation or hypnotic component [52,53]. In this study, the depth of anesthesia was adjusted based on the BIS value, but it is known that this responsiveness varies depending on the anesthetic agents, and those findings may affect the CBF results. In future studies, a better accuracy may be achieved using PPG or CSI, which do not differ in responsiveness depending on the anesthetic agents.

Other biomarkers, including biochemical and neuroimaging biomarkers, are also examined in patients with cognitive decline. The S100B protein, neuron-specific enolase, and tau protein levels were increased in serum and cerebrospinal fluid (CSF) in patients undergoing surgery [54]. However, no significant differences were observed in CSF amyloid beta (Aβ) and tau protein levels between isoflurane and propofol anesthesia [55]. The CSF soluble triggering receptor-expressing myeloid cells 2 (sTREM2) was significantly associated with the presence or absence of Aβ and tau protein on positron emission tomography (PET) imaging [56]. Although there are currently few confirmed results, future studies may be able to use these biomarkers to achieve multimodal analysis.

No changes in the TOI were found herein in either the dementia-positive or dementia-negative group. Although the TOI does not change as rapidly as arterial oxygen partial pressure and SpO_2_, a study reported an increase in the TOI with oxygen administration [39]. In this study, TOI may not have changed as oxygen was administered at 100% during anesthesia induction and 33% during anesthesia maintenance.

The highest MAP value after the induction of anesthesia was significantly higher in the dementia-positive group (MAP 3: median, 98 mmHg) than in the dementia-negative group (MAP 3: median, 86 mmHg). The highest BIS values were also significantly higher in the dementia-positive group (BIS 2 and 3: median values, 74 and 67, respectively; non-dementia group: median values, 55.5 and 58, respectively). These values in the dementia-positive group may have increased due to stimulation such as anesthesia induction and tracheal intubation because the depth of anesthesia was somewhat shallow in the dementia-positive group. Because anesthetics and narcotics can excessively decrease blood pressure and BIS values in elderly patients, we used limited amounts [41].

As the dementia grades (FAST and CDR) of the patients with dementia did not deteriorate after anesthesia, we concluded that the nTHI values decreased, but there was no decline in cognitive function among patients with severe dementia at FAST stages 6e to 7c and/or CDR indicating severe dementia. However, each category of dementia grade (FAST and CDR) has a certain range of symptoms. A subtle cognitive decline might result in the patient continuing to be classified in the same grade, due to the weak detection capability of FAST and CDR [36].

This study shares some limitations with those of our previous reports [34,35]. One limitation is that the probes for measuring the brainwaves (BIS) and CBF (NIRO^®^-200NX) had to be placed on the forehead, and despite measuring the BIS and CBF at the forehead, the conditions at other locations in the brain were not evaluated. Some reports showed differences in blood flow changes in different parts of the brain depending on the anesthetics used; more detailed measurements at multiple sites of the brain may thus be necessary [41]. In the present study, we also tried to adjust the types and volumes of anesthetics and opioids in both groups to maintain the MAP, BIS, SpO_2_, and etCO_2_ values within a preferable range. However, the complete matching of the drugs used and their volume was not possible. Although the anesthesia method used in this study is thought to have a small effect on CBF in both groups, it may be an issue that should be considered when discussing the results. Although this study satisfied the requirements of the statistical sample size calculation, the sample size was small, which is another limitation. Future large-scale studies should evaluate cerebral and cognitive function during and after anesthesia.

## 5. Conclusions

The present study showed that when anesthesia was slowly induced while controlling the MAP, BIS, SpO_2_, and etCO_2_ values within a preferable range, the nTHI value of elderly patients with severe dementia was 0.60 (a decrease of 40%); however, an nTHI value of 0.79 (a decrease of 20%) was observed in elderly patients with no cognitive decline. These findings indicate that the nTHI value decreases significantly in elderly patients with severe dementia. In addition, no change was observed in the TOI.

## Figures and Tables

**Table 1:** Characteristics of patients (*n* = 26).

	Patients With Dementia (*n* = 13)	Patients Without Dementia (*n* = 13)	*p* Value	Mann–Whitney U Test
Age (years)	70 (65–84)	68 (54.5–77)	0.113	53.5
Sex (male/female)	6/7	10/3	0.186	58.5
Height (cm)	157 (147.8–168.0)	158.4 (156.4–165)	0.724	77.0
Weight (kg)	49 (43.5–59.7)	50.8 (50–56.1)	0.579	73.0
Body mass index	20.4 (19.4–23.1)	20.4 (18.9–22.6)	0.880	81.5
Treatment time (min)	177 (159.5–195)	147 (122–174)	0.029	42.0
Anesthesia time (min)	230 (215–258)	196 (161.5–234.5)	0.016	38.0
Time between induction and intubation (min)	17 (15–21)	12 (9–18.5)	0.125	54.5
Time between intubation and treatment (min)	17 (14–20)	16.0 (14.5–25.5)	0.960	83.0
**Induction**				
Fentanyl (μg)	75 (75–75)	75 (50–100)	0.949	21.0
Remifentanil (μg/kg/min)	0.2 (0.1–0.3)	0.20 (0.2–0.35)	0.582	6.5
Sevoflurane (%)	5.0 (3.5–5.0)	5.0 (5.0–5.0)	0.439	13.5
**Maintenance**				
Fentanyl (μg)	25 (25–50)	50 (25–50)	0.517	7.5
Remifentanil (μg/kg/min)				
High	0.10 (0.10–0.15)	0.15 (0.11–0.19)	0.261	28.0
Low	0.06 (0.05–0.07)	0.1 (0.035–0.1)	0.340	30.5
Sevoflurane (%)				
High	1.0 (1.0–1.5)	1.0 (1.0–1.5)	0.362	66.5
Low	0.5 (0.5–1.0)	1.0 (1.0–1.0)	0.064	48.5

## Data Availability

The datasets presented in this study are available upon reasonable request from the corresponding author.

## Supplementary Materials

The following supporting information can be downloaded at: https://www.mdpi.com/article/10.3390/jcm14196692/s1, Table S1: Patient’s vital sign data; Table S2: The data of the normalized tissue hemoglobin index (nTHI) and tissue oxygenation index (TOI).
